# An Efficient and Adaptive Mutual Authentication Framework for Heterogeneous Wireless Sensor Network-Based Applications

**DOI:** 10.3390/s140202732

**Published:** 2014-02-11

**Authors:** Pardeep Kumar, Mika Ylianttila, Andrei Gurtov, Sang-Gon Lee, Hoon-Jae Lee

**Affiliations:** 1 Centre for Wireless Communication, University of Oulu, P.O. Box 4500, FI 90014, Finland; E-Mail: mika.ylianttila@ee.oulu.fi; 2 Helsinki Institute of Information Technology, P.O. Box 15600, Aalto 00076, Finland; E-Mail: gurtov@hiit.fi; 3 Department of Ubiquitous-IT, Dongseo University, San 69-1, Jurye-2-Dong, Sasang-Gu, Busan 617-716, South Korea; E-Mails: nok60@dongseo.ac.kr (S.-G.L.); hjlee@dongseo.ac.kr (H.-J.L.)

**Keywords:** security, mutual authentication, key establishment, node privacy, wireless sensor networks

## Abstract

Robust security is highly coveted in real wireless sensor network (WSN) applications since wireless sensors' sense critical data from the application environment. This article presents an efficient and adaptive mutual authentication framework that suits real heterogeneous WSN-based applications (such as smart homes, industrial environments, smart grids, and healthcare monitoring). The proposed framework offers: (i) key initialization; (ii) secure network (cluster) formation (*i.e.*, mutual authentication and dynamic key establishment); (iii) key revocation; and (iv) new node addition into the network. The correctness of the proposed scheme is formally verified. An extensive analysis shows the proposed scheme coupled with message confidentiality, mutual authentication and dynamic session key establishment, node privacy, and message freshness. Moreover, the preliminary study also reveals the proposed framework is secure against popular types of attacks, such as impersonation attacks, man-in-the-middle attacks, replay attacks, and information-leakage attacks. As a result, we believe the proposed framework achieves efficiency at reasonable computation and communication costs and it can be a safeguard to real heterogeneous WSN applications.

## 1. Introduction

Wireless sensor networks (WSNs) are intelligently integrated with the Internet and becoming popular sources of data fusion in real-world mission-critical applications (e.g., smart homes, healthcare, power plants, homeland security, smart buildings, *etc.*). In a sensor network, numbers of wireless sensors collect and (partially) process the application raw data anytime and wirelessly forward the collected data through relay nodes to anywhere (e.g., a remote server). In practice, a real WSN application consists of heterogeneous sensor nodes (*i.e.*, low-capacity nodes and high-capacity nodes) [1–7]. A low-capacity node, *i.e.*, L-node, is a resource-constrained node (e.g., Telos [8], MicaZ [9], *etc.*) that has low bandwidth, less computation power, less memory, and low battery power [3]. A high-capacity node, *i.e.*, H-node, is a much more resource-rich node (e.g., stargate node [10]) that has high transmission range and directional antenna, more computation power and memory. Recently, many researchers have shown the heterogeneous sensor networks are very suitable for real-time applications, and have better performance, reliability, scalability, transparency, load-balancing, network life-time, and cost-efficiency [11–15] *etc.* Thus, heterogeneous sensor networks are more efficient and practical in real-time applications.

The deployment of heterogeneous WSNs promises reliable data transmission, scalability, load-balancing and application efficiency [12–15]; however, it bring a plethora of security related issues (such as mutual authentication and session-key establishment, confidentiality, and message freshness) that must be introduced at the application design time. The resource-hungry sensor nodes are deployed in open environments, where they are susceptible to attacks by global adversaries using compromised nodes. Furthermore, there is no denying that wireless channels are more vulnerable than wired networks. In a mission-critical application, if the technology fails due to the lack of strong and adequate security then it will affect (people's) day-to-day life or damage its long-term application's viability. Therefore, to protect WSNs from unauthorized access (e.g., the compromised nodes and/or the global adversary), all the sensor nodes in a network should perform the following: (i) mutual authentication to establish a common trust; (ii) two communicating nodes should establish a dynamic session key after performing the authentication; and (iii) all the wireless data must be secured (*i.e.*, confidential) while in transit. Thus, the mission-critical applications require an efficient and adaptive mutual authentication framework that can establish a common trust within the network and protect the network from unauthorized access and security threats.

During the last decade, a number of security protocols have been proposed for homogeneous WSNs [16–26], and for heterogeneous WSNs [27–38]. Indeed, each protocol has advantages and disadvantages. However, in the existing researches (see Section 3), the focus is on unilateral authentication (*i.e.*, one-way authentication), where each scheme exploits the respective key management system. The main disadvantage of one-way authentication is that a node is not able to know whether it is connected with a legal entity or a fake one; therefore, the mutual trust between the two communicating parties is zero. In addition, to perform node authentication in key management schemes, there is no 100% guarantee that a shared key will be found. Due to the lack of mutual authentication in the network devices, the dynamic session key has the lowest priority. Moreover, to perform the authentication between two nodes/devices, high numbers of keys are suggested to a sensor node in [28,30,37]. However, the high numbers of keys may pose the Sybil threats to the applications if a node is compromised by an adversary. In [31–33,35], a sensor node required a smaller number of keys to perform the authentication, but authors did not care for strong mutual authentication and session key establishment, node privacy, and message confidentiality and freshness. Therefore an efficient and adaptive mutual authentication framework remains a challenge for real WSN applications.

To address mutual authentication in WSNs-based applications, this paper introduces an efficient and adaptive mutual authentication framework that exploits the features of symmetric key cryptography and provides strong mutual authentication and strong key establishment, message confidentiality, node identity and location privacy, and message freshness. The proposed scheme makes use of the pre-deployment location of sensors nodes which improve the application processes and operational efficiencies [16,28,32]. The proposed framework is very simple and performs the following tasks:

- Firstly, sensor nodes (L-sensor and H-sensor) obtain the required keys from an offline key server, as in [30–33].
- Secondly, a secure network (cluster) formation takes place where the L-sensor and H-sensor mutually authenticate each other and establish a strong dynamic session key.
- Thirdly, a key revocation mechanism copes with the case of compromised L-sensor nodes, if found in the network.
- Finally, a new L-sensor node addition technique facilitates the node scalability to the application and supports maximum network size.

This paper further demonstrates the correctness of the proposed framework using Burrows, Abadi, and Needham (BAN) logic, which is a quite popular logic for verifying mutual authentication and session-key establishment schemes [39,40]. The security analysis shows that the proposed scheme offers strong safeguards against possible security attacks such as impersonation attacks, man-in-the-middle attacks, replay attacks and information-leakage attacks.

The rest of the paper is structured as follows: Section 2 describes the system model, threat model and design goals. Section 3 discusses the related work and Section 4 introduces the detailed design of proposed scheme for real WSNs. Section 5 proves the correctness using BAN logic. Sections 6 and 7 shows the comparative analysis and the discussion, respectively. Finally, Section 8 presents the conclusions.

## 2. System Model, Threat Model and Design Goals

### 2.1. System Model

**Figure 1.**
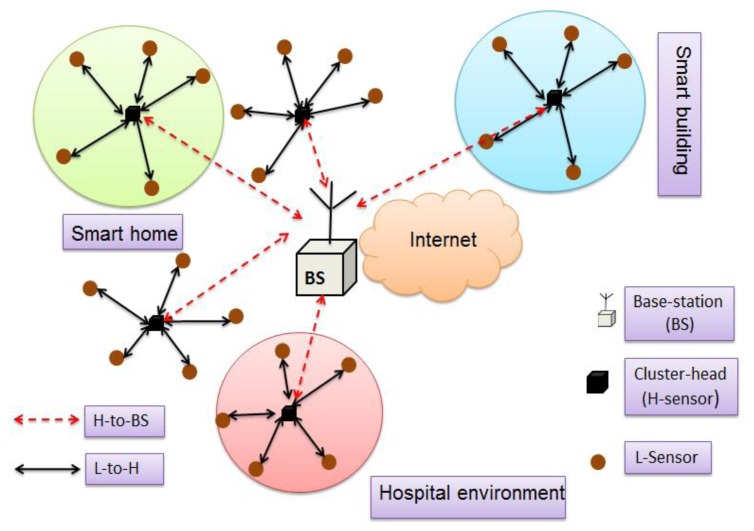
A system model for distributed WSN applications.

It is widely accepted that clustered or distributed heterogeneous sensor networks can intelligently perform with network efficiency, operational performance, and long-lasting network life-times [27–35]. Figure 1 depicts a model of a distributed WSN system, which is mainly composed of sensor nodes (L-sensors), cluster-heads (H-sensors), and a base-station (BS). This distributed system model is very suitable for mission-critical monitoring applications where sensors need to be deployed strategically, as suggested in [1,2,5,7,41,42]. Some of these applications are smart buildings, hospital environments, smart homes, nuclear power plants, gas-plants, and so on.

In a heterogeneous clustered approach, as depicted in Figure 1, the L-sensors are resource-constrained devices (low power, short communication range, limited memory, and less computation power); while H-sensors are equipped with tamper-resistance and have more resources (such as high power, large communication ranges, large memory capacity and computation power). The L-sensors are strategically deployed in a cluster and each cluster is controlled by a cluster-head (H-sensor). The L-sensors simply sense the environment ambient data and forward it to the H-sensors and *vice versa* (*i.e.*, cluster-heads can also request sensors' data). It is assumed that the H-sensor can perform complex operations on the sensor data, and using longer radio it can directly communicate to the base-station. The base-station (BS) is a powerful node and it has unlimited resources. The base-station may be a remote server and it may be connected to the outer-world using the high-speed Internet.

In [32,33,43], the authors have suggested that generally L-sensors do not need to share their data among themselves, hence connectivity between two L-sensors are not required, as found in real-time applications (e.g., volatile organic compound monitoring [1], healthcare [2], plant monitoring [5], and hazardous site monitoring [7]). Our network model also follows the same assumption, so no data sharing is allowed between two L-sensors. In the other words, L-sensors can directly communicate to the H-sensor and *vice versa*, while the H-sensor is directly connected to the BS.

### 2.2. Threat Model

Many of the schemes [27,32,33,35] have assumed that an adversary is not present during the network (cluster) formation. However, this is not practical in real WSNs. Based on the above system model (Figure 1), we have assumed that an attacker is active from the beginning of the WSN deployment. The adversary might deploy malicious nodes into the deployment terrain and try to misguide the network functionality. Further, a global attacker can easily monitor the wireless traffic of a WSN, and can capture the wireless packets. He/she can gain much useful sensor information from the captured packet (such as node IDs and sensor location, *etc.*), and can modify a packet information (IDs, location, *etc.*) while a packet is in transit. More importantly, an attacker can intentionally capture a node and obtain all its cryptographic keys. As a result, node capturing attacks are very harmful for the network if high numbers of cryptographic keys are stored on a node.

### 2.3. Design Goals

Based on the system and attack model, we mainly focus on the following security goals: since the L-sensors are not trustworthy, *initializing* necessary keys facilitates strong security. To maintain the mutual trust between the network devices (sensors, cluster-head and base-station), devices must perform *mutual authentication* and establish a *dynamic session key* before establishing the session, so that both the nodes/devices can confirm their legitimacy. In critical applications (homeland security, healthcare, smart grids), nodes' identities are very important, thus, nodes' identities (*i.e.*, *node privacy*) should be kept safe and private [44–46]. To protect the wireless communication from illegal eavesdropping and interception all the wireless messages should be secured (*i.e.*, *confidential*), so that a global adversary cannot read, monitor, intercept, or alter the wireless messages. In practice the L-sensors are easily compromised, so then a *key revocation* mechanism can play an important role to protect from any future misleading actions from a compromised node. Furthermore, a *node addition* feature provides scalability to the network.

## 3. Literature Survey

A number of anonymous security schemes have been proposed for heterogeneous WSNs in recent years, and they present different types and/or levels of security protection at different costs. In this section, we present the existing schemes that address the issue of authentication in heterogeneous WSNs. We have divided this section into two: (i) unilateral; and (ii) mutual authentication.

### 3.1. Unilateral Authentication

Du *et al.* [28] proposed an asymmetric pre-distribution key management scheme which exploits the deployment knowledge, and composed of three phases: cluster formation, asymmetric pre-distribution key management and H-sensor based pairwise key setup phase. To perform the security services (e.g., node authentication), each H-sensor is preloaded with *M* keys with their corresponding key identities (*IDs*) from a key pool. Likewise, each L-sensor is preloaded with *l* keys with the corresponding *ID* keys from a key pool. However, during the cluster formation no unilateral/mutual authentication is allowed between the two L-sensors and the H-sensors and hence an insecure cluster formed without verifying the node identities.

Maala *et al.* [31] presented a HERO protocol. HERO requires less key storage, especially for L-sensors as compared to [28]. In this scheme, a gateway initiated secure tree is established using the L-sensor and the H-sensor. Each attached node is authenticated through a message authentication code (MAC) (*i.e.*, *{ID_i_*,*MAC*(*K1*, *ID_i_*), *MAC*(*K2*, *ID_i_*)*}*), which is computed over its identifier (*ID*) and each key (*K*) of its key ring. Although HERO facilitates unilateral authentication and integrity, key revocation, and new node addition, it does not address the imperative security requirement (*i.e.*, the mutual authentication and dynamic session key establishment).

Jolly *et al.* [32] proposed a low energy key management (LEKM) protocol where each L-sensor is preloaded with two keys (one key is shared with the H-sensor and another key is shared with the base-station). The main drawback of LEKM is that when the L-sensor communicates with the H-sensor, it performs (a weak) one-way authentication (*i.e.*, unilateral authentication) and leaks sensitive privacy information (e.g., node identity). Another drawback of LEKM is that it does not establish a dynamic session-key, which is a paramount security requirement for WSN applications. In addition, Cheng and Agrawal have demonstrated the security weaknesses of LEKM [33].

In [37] the authors proposed a secure clustering and symmetric key establishment scheme which is based on public key cryptography (PKC). In this scheme, each L-sensor is preloaded with its public and privacy keys, and the public keys of all the H-sensors. The scheme provides many security services, such as secure clustering, symmetric key establishment, unicast authentication, and message freshness. Moreover, the scheme leverages on PKC based elliptic curve digital signature algorithm (ECDSA), which is still expensive for the L-sensor nodes [20].

### 3.2. Mutual Authentication

Traynor *et al.* [30] presented a LIGER hybrid security mechanism that has two sub-schemes: LION and TIGER. Authors assumed that each L-sensor is preloaded with *X* keys (e.g., 30) along with key identifiers and the H-sensor is preloaded with a minimum *Y* keys (e.g., 711) with keys identifiers (in both schemes). LION is a standalone key mechanism where an L-sensor learns its neighbors through a (one-way) *Hello* message and then establishes keys (*i.e.*, no mutual authentication), whereas in TIGER, two L-sensors authenticate each other and establish a secure session key using a key distribution center (KDC). TIGER could be suitable for smart buildings or factories where L-sensors gather data from the environment, but it requires that the KDC always be online.

In [33], Cheng and Agrawal proposed an improved key distribution mechanism compared to [32]. The proposed protocol is composed of three phases, namely, key pre-distribution, inter-cluster pairwise establishment and intra-cluster pairwise establishment. Each L-sensor has two pre-installed keys. The H-sensor has a shared key with the base-station and, in addition, it has two polynomials, which are shared with neighboring H-sensors. In the inter-cluster pairwise key establishment phase, two cluster-heads exchange their identities and perform mutual authentication to establish a pairwise static key. Similarly, in the intra-cluster key establishment phase, a static pairwise key is established after performing the node authentication. The main drawback of [33] is that the authors did not consider dynamic session keys for particular sessions. In addition, Paterson-Stinson demonstrated that IDKM is susceptible to two types of attacks (interpolation attacks and reconstruction attacks) [47]. Moreover, in the intra-cluster pairwise key establishment phase, finding a shared key may take a long time if the intended H-sensor is quite far away from the L-sensor, and thus the communication costs would be expensive.

In 2009, Huang proposed a novel access control protocol [48]. The author performed mutual authentication and key establishment for two neighboring nodes, and claimed that the proposed scheme is robust against masquerade and replay attacks. Unfortunately, Kim and Lee pointed out that contrary to the claims in [48], the Huang protocol is vulnerable to replay and masquerading attacks and provides only unilateral authentication [49].

In addition, in [49] Kim and Lee proposed an enhanced novel access control protocol (ENACP) over WSNs, and claimed that their protocol safeguards against masquerading attacks and forgery attacks and supports secure connectivity. Zeng *et al.* have shown that ENACP is vulnerable to new node masquerading attacks and legal node masquerading attacks [50]. In addition, the schemes proposed in [48,49] are based on ECC that requires expensive computation overhead.

## 4. Proposed Mutual Authentication Framework

In order to achieve the security goals (see Section 2.3) for distributed WSN applications, this section presents the detailed design of our proposed mutual authentication framework. The proposed scheme consists of four phases, namely, key generation and initialization, sensor deployment and secure network formation, key revocation, and new node addition phase. Before starting the scheme, we have to make some assumptions suited to real-time WSN applications, as follows:

- A base-station is a trusted entity. Each L-sensor can directly communicate with the H-sensor and *vice versa*; and both sensors (L-sensor and H-sensor) are location aware and static (no mobility).
- We have assumed that the cryptosystems used are strong enough to ensure that air messages cannot be decrypted without having the secret keys.

Table 1 lists the notations and descriptions used throughout the rest of paper.

### 4.1. Key Generation and Initialization Phase

*L-sensors*: The base-station (BS) generates an offline key pool (*KPL*) of keys (*KL_i_*) with their corresponding key indexes (*Kidx_Li_*). Note that the length of the key pool depends on the number of L-sensors to be deployed in the application and there are no common keys in the pool (*i.e.*, *KL_i_*∩*KL_j_* = *Ø and i* ≠ *j*). Upon generating the key pool, the base-station initializes a unique key with its key index to each L-sensor. In addition, the base-station assigns a unique identity (*LID_i_*) to each L-sensor and the location (*Loc_i_*) where it will be deployed (as shown in real-time volatile organic compound monitoring [1,7]) along with its cluster-head identity (*HID_i_*). Each L-sensor has symmetric cryptosystems, e.g., Skipjack, a hash function (MD5 or SHA-1) [35,51,52], and a random number generator [53].

*H-Sensor*: The BS generates another offline key pool (*KPH*) of keys (*KH_i_*) with their corresponding key indexes (*Kidx_Hi_*). The length of the key pool depends on the required number of cluster-heads and no keys are common in the pool (*i.e.*; *KH_i_*∩*KH_j_* = *Ø and i* ≠ *j*). The base-station assigns a unique key with its key index and identity (*HID_i_*) to each H-sensor. Since an H-sensor is a resource-rich (high storage) and tamper-proof device, it is preloaded with its member nodes identities (*LID_i_*), locations (*Loc_i_*) and keys (*KL_i_*) with their indexes (*Kidx_Li_*). Each H-sensor also assigned its neighboring H-sensors identities (*HID_i-1_*) and the corresponding keys (*KH_i-1_*) with their indexes (*Kidx_Hi-1_*). In addition, each H-sensor has the identical (as the L-sensors) symmetric cryptosystems, and has the identity of the base station (*i.e.*; *ID_BS_*). Finally, the BS maintains a table that keeps the records all the H-sensors (*HID_i_*), L-sensors (*LID_i_*), locations of sensors and all assigned keys with their indexes.

### 4.2. Sensor Deployment and Secure Network Formation

As shown in Figure 1, sensors should be deployed, strategically, as in [1,2,4,7,41,42,54,55]. The H-sensor communicates with the L-sensor (in an *ad hoc* manner) using a wireless link (*i.e.*, black solid lines in Figure 1), which is called the “H-To-L” communication link [32,33]. Moreover, whenever an H-sensor wants to disseminate sensor data to the BS (*i.e.*, which could be application dependent), it can wirelessly communicate to the BS using a long-haul transmission (*i.e.*, red dotted lines in Figure 1), which is called “H-To-BS” communication links. Upon deploying the sensors, a secure network formation starts, as follows:

#### 4.2.1. H-To-L Communication Link

The H-sensor broadcasts a *hello* message that shows its own presence to the cluster members (*i.e.*, L-sensors). If the L-sensor does not receive a *hello* message from the H-sensor within the specified time, then L-sensor broadcasts its own *hello* message to the H-sensor. The procedure is as follows:

1. H-sensor generates a *hello* message (*i.e.*, *B* = *h*(*HID_i_*) ) and sends it to the L-sensors.
2. Upon receiving the *hello* message, L-sensor computes: *B′* = *h*(*HID_i_*) and verifies if *B′* = *B*, if yes then the H-sensor is a legitimate entity and it goes to the next steps; otherwise, it waits for a legitimate request. Thereafter, it computes: *C* = *E_KLi_[LID_i_*, *Loc_i_*, *R0]*, here *R0* is a dynamic secret number, which is generated by the L-sensor. Now the L-sensor sends *C* and *Kidx_Li_* (index of key *KL_i_*) to the H-sensor.

Upon receiving the messages *i.e.*, *C* and *Kidx_Li_* from an L-sensor, H-sensor performs the following and checks whether the L-sensor is a legitimate sensor or not:

3. Get the corresponding key (*KL_i_*) of *Kidx_Li_* from its members (L-sensors) key lists. Decrypts sub-message *C* using the *KL_i_* to obtain LID_i_*, Loc_i_* and R0*. Verifies LID_i_* = *LID_i_*, and Loc_i_* = *Loc_i_*, if yes then the L-sensor is a legitimate node and goes to the next steps; otherwise the L-sensor is a fake entity and the system aborts. Moreover, the H-sensor keeps the value of *R0* in its records which will protect it from replay attacks.
4. Computes *X* = (*KL_i_ ⊕ R0*), *Q* = *h*(*R0*‖*R1*‖*LID_i_*‖*HID_i_*), here, *R1* is a dynamic secret number which is generated by the H-sensor. Now, it generates a message *M* = *E_X_[LID_i_*, *HID_i_*, *R0*, *Q]* and sends <*M*, *R1*> to the L-sensor.

After receiving message from the H-sensor, the L-sensor performs the following actions:

5. Computes *X* = (*KL_i_ ⨁ R0*), and decrypts *M* using *X* to obtain LID_i_*,HID_i_*, R0*, and Q*.
6. Verifies LID_i_* = *LID_i_*, HID_i_* = *HID_i_*, and R0* = *R0*; if these checks pas*s* correctly, it means that both the entities are legitimate; otherwise not. Now, it computes *Q* and verifies Q* = *Q*, if true, then a session key is established.

**Figure 2.**
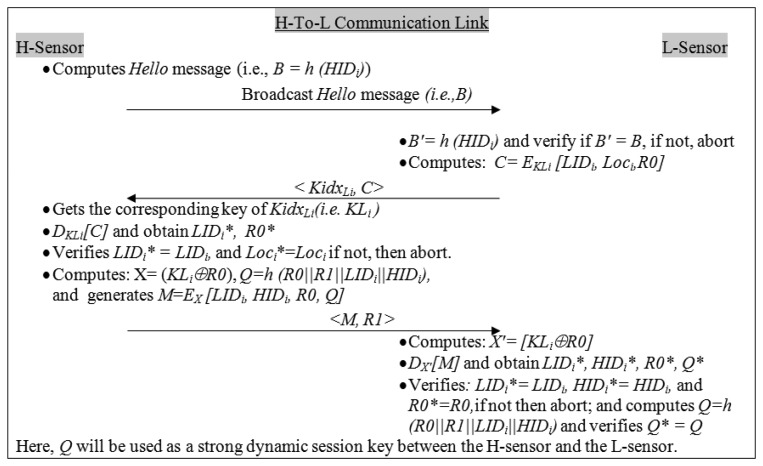
The message flow of an H-To-L link.

Here, Q* is a strong dynamic session key between the H-sensor and the L-sensor. Hence a secure network formation is completed for the H-To-L communication link. The H-To-L link mutual authentication flow is shown in Figure 2.

#### 4.2.2. H-To-BS Communication Link

**Figure 3.**
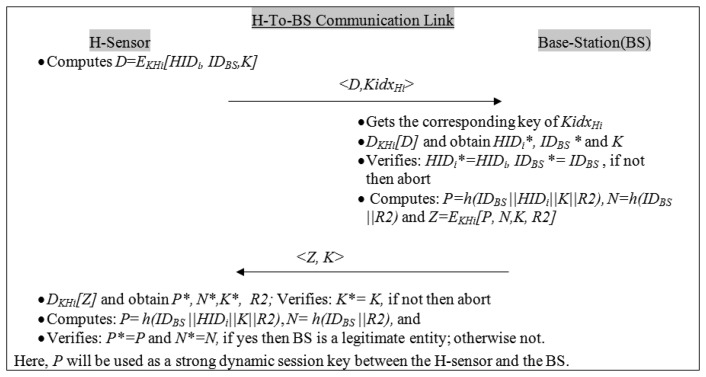
The messages flow of H-To-BS link.

This phase is invoked whenever an H-sensor/cluster-head wants to communicate with the base-station. In this link, a secure link is set up as follows:

1. H-sensor sends a message <*D*, *Kidx_Hi_*> to the BS. Here, *D* = *E_KHi_[HID_i_*, *ID_BS_*, *K]*, and *K* is a H-sensor's dynamic secret number, and *Kidx_Hi_* is the key index (*KH_i_*).
2. Upon receiving the message <*D*, *Kidx_Hi_*>, the BS gets the corresponding key of *Kidx_Hi_* and decrypts the message *D* to obtain HID_i_*, *ID_BS_* * and *K*. Checks HID_i_* = *HID_i_*, ID_BS_* = *ID_BS_* with its own stored records. If it holds, then it goes to the next steps; otherwise, it aborts the system. Moreover, BS keeps the value of *K* in its records which will protect it from replay attacks.
3. Computes *P* = *h* (*ID_BS_*‖*HID_i_*‖*K*‖*R2*), *N* = *h*(*ID_BS_*‖*R2*) and *Z* = *E_KHi_[P*, *N*, *K*, *R2]*; sends the message <*Z*, *K*> to the H-sensor. Here *R2* is a dynamic secret number that is generated by the BS.
4. After receiving the message <*Z*, *K*> from the BS, the H-sensor decrypts the message *Z* to obtain P*, N*, K*, and *R2*. Verifies K* = *K*, if not then it terminates the system. Otherwise, it computes *P* = *h*(*ID_BS_*‖*HID_i_*‖*K*‖*R2*), *N* = *h*(*ID_BS_*‖*R2*), and verifies P* = *P* and N* = *N*. If it holds, it means the BS is a legitimate entity. Now, *P* will be used as a strong dynamic session key between the H-sensor and the base station. Moreover, H-sensor keeps the value of *R2* in its records, which will protect it from replay attacks. The H-To-BS link mutual authentication flow is depicted in Figure 3.

### 4.3. Key Revocation Phase

The key revocation phase is triggered when an L-sensor is compromised by an adversary. The adversary can extract all the information stored on an L-sensor and start misleading the network (*i.e.*, also known as misbehavior). Assume that the H-sensor detects the misbehavior of a compromised link (*i.e.*, L-sensor) by using the scheme described in [56]. After detecting the misbehavior of an L-sensor, the H-sensor generates a revocation message that contains information about the compromised link (*i.e.*, *LID_i_*, *KL_i_*, *Kidx_Li_*, *Loc_i_*) and securely sends the revocation message to the BS. By doing so, the BS will become aware of the compromised L-sensor and updates its own list. Moreover, if anyhow, a compromised link is not detected by the H-sensor, it will not affect the non-compromised links because there is no direct communication between two L-sensors. Hence, the non-compromised L-sensors are secure and continue to work properly.

### 4.4. New L-Sensor Addition Phase

Secure scalability is the most important factor for the network success. However, the addition of a new node is a challenging task due to two main reasons: (i) the new node could be a malicious node; or (ii) the new node could be a clone of a compromised node. In the proposed scheme, a new node addition is very simple because the new node addition request message is securely forwarded by the base station to the designated H-sensor. Suppose a new L-sensor needs to be added into the network; the BS first loads the necessary parameters into the new L-sensor. Now it will securely pass the new L-sensor's information and parameters to the designated H-sensor where the L-sensor needs to be deployed. Thereafter, the L-sensor has to perform the same procedures (*i.e.*, recall 4.2.1 (H-To-L communication link)); by doing this, the new L-sensor will become a legitimate member of the H-sensor's network.

## 5. Correctness Verification/Proof

This section verifies and ensures the correctness of the proposed scheme, *i.e.*, authentication and key-establishment using BAN logic [39]. The BAN logic was proposed by Burrows, Abadi, and Needham, and is relatively simple to use to ensure the proof-of-correctness of authentication and key-establishment protocols. It is a logic of belief (*i.e.*, trustworthiness). We demonstrate the beliefs of trustworthiness of involved parties in the proposed scheme. The notations and rules for verification are introduced in [39]. In the proposed framework, the main principals are the following: BS, H-sensor and L-sensor. The goal of verification is to verify the correctness of a dynamic session key after performing the mutual authentications between the two involved principals. In order to verify the correctness, we will first verify the H-To-L communication link and then the H-To-BS communication link, as follows:

### 5.1. H-To-L Communication Link

To perform the formal verification, the following postulates need to be considered:

- *L-sensor* ***believes*** $L‐\text{sensor}\overset{Q}{↔}H‐\text{sensor}$
- *L-sensor* ***believes*** *H-sensor* ***believes*** $L‐\text{sensor}\overset{Q}{↔}H‐\text{sensor}$
- *H-sensor* ***believes*** $L‐\text{sensor}\overset{Q}{↔}H‐\text{sensor}$
- *H-sensor* ***believes*** *L-sensor* ***believes*** $L‐\text{sensor}\overset{Q}{↔}H_{i}$

Now, H-To-L communication link messages (see Figure 2) should be re-arranged from the generic form to the idealized form, as follows:

Generic messages form
  Msg 1: *H-sensor* ➔ *L-sensor*: <*B*> (*here*, *B*= *h*(*HID_i_*))
  Msg 2: *L-sensor* ➔ *H-sensor*: <*Kidx_Li_*, *C*> (*here*, *C*= *E_KLi_[LID_i_, Loc_i,_ R0]*)
  Msg 3: *H-sensor* ➔ *L-sensor*: <*M,R1*> (*here*, *M* = *E_X_[LID_i_, HID_i_, R0, Q]*)
Idealized messages form
  Msg 1: *H-sensor* ➔ *L-sensor*: *{ h*(*HID_i_*)*}*
  Msg 2: *L-sensor* ➔ *H-sensor*: *{LID_i_, Loc_i,_ R0}_KLi_*, *Kidx_Li_*
  Msg 3: *H-sensor* ➔ *L-sensor*: *{LID_i_, HID_i_, R0, Q} _X_*, *R1*
*Session Key Q*= *h*(*R0*‖*R1*‖*LID_i_*‖*HID_i_*)

To further analyze the H-To-L link using BAN logic, the following hypotheses are also needed:

(*A1*) *H-sensor* ***believes*** $L‐\text{sensor}\overset{\text{KLi}}{↔}H‐\text{sensor}$
(*A2*) *L-sensor* ***believes*** $H‐\text{sensor}\overset{\text{KLi}}{↔}L‐\text{sensor}$
(*A3*) *H-sensor* ***believes*** (*L-sensor* ***controls*** *LID_i_*)
(*A4*) *L-sensor* ***believes*** (*H-sensor* ***controls*** HID_i_)
(*A5*) *L-sensor* ***believes*** $H‐\text{sensor}\overset{Q}{↔}L‐\text{sensor}$
(*A6*) *H-sensor* ***believes*** $L‐\text{sensor}\overset{Q}{↔}H‐\text{sensor}$
(*A7*) *H-sensor* ***believes*** $H‐\text{sensor}\overset{Q}{↔}H‐\text{sensor}$
(*A8*) *L-sensor* ***believes*** (*H-sensor* ***controls*** $H‐\text{sensor}\overset{Q}{↔}L‐\text{sensor}$)
(*A9*) *H-sensor* ***believes*** *L-sensor* ***fresh*** (*R0*)
(*A10*) *L-sensor* ***believes*** *H-sensor* ***fresh*** (*R1*)

Based on above assumptions and [39] rules, we present the H-To-L communication link formal proof, as follows:

H-To-L correctness proof
  Msg 1: *H-sensor* ➔ *L-sensor*: *{h*(*HID_i_*)*}*
    (*B1*) *L-sensor sees {h*(*HID_i_*)*}*//*by Seeing rule*
    (*B2*) *H-sensor* ***believes*** *L-sensor* ***said*** *{LID_i_*, *Loc_i,_ R0} _KLi_*, *Kidx_Li_*//*Message-meaning rule*
  Msg 2: *L-sensor* ➔ *H-sensor*: *{LID_i_, Loc_i,_ R0}_KLi_, Kidx_Li_*
    (*B3*) *H-sensor* ***Sees*** *{LID_i_, Loc_i,_ R0} _KLi_, Kidx_Li_*//*Seeing rule*
    (*B4*) *H-sensor believes* (*L-sensor* ***controls*** *LID_i_*)//*A3, B3, Control rule*
    (*B5*) *H-sensor* ***believes*** *L-sensor* ***fresh*** (*R0*)//*A9, Fresh rule*
    (*B6*) *H-sensor* ***believes*** $L‐\text{sensor}\overset{X}{↔}H‐\text{sensor}//\text{Message}‐\text{meaning}\;\text{rule}$
    (*B7*) *H-sensor* ***believes*** *L-sensor* ***said*** *{LID_i_, HID_i_, R0, Q} _X_*
  Msg 3: *H-sensor* ➔ *L-sensor*: *{LID_i_, HID_i_, R0, Q}_X_, R1*
    (*B8*) *L-sensor* ***believes*** $H‐\text{sensor}\overset{X}{↔}L‐\text{sensor}//\text{Message}‐\text{meaning}\;\text{rule}$
    (*B9*) *L-sensor* ***sees*** *{LID_i_, HID_i_, R0, Q} _X_*//*B8, Seeing rule*
    (*B10*) *L-sensor* ***believes*** (*H-sensor* ***controls*** *HID_i_*)//*A4, B9, Control rule*
    (*B11*) *L-sensor* ***believes*** (*H-sensor* ***controls*** $H‐\text{sensor}\overset{Q}{↔}L‐\text{sensor}$)//*A8, B9*
    (*B12*) *L-sensor* ***believes*** $H‐\text{sensor}\overset{Q}{↔}L‐\text{sensor}$

By doing so we have formally verified the H-To-L communication link goals, *i.e.*, *A5* and *B12* establish the secure session-key (*i.e.*, *Q*) between the L-sensor and the H-sensor. *A4*, *A5*, *B4*, *B10* and *B12* verify the mutual-authentication between the H-sensor and the L-sensor.

### 5.2. H-To-BS Communication Link

Similarly, to perform the formal verification of the H-To-BS communication link (see Figure 3), the following postulates need to be considered:

- *BS* ***believes*** $H‐\text{sensor}\overset{P}{↔}BS$
- *BS* ***believes*** *H-sensor* ***believes*** $BS\overset{P}{↔}H‐\text{sensor}$
- *H-sensor* ***believes*** $BS\overset{P}{↔}H‐\text{sensor}$
- *H-sensor* ***believes*** *BS* ***believes*** $H‐\text{sensor}\overset{P}{↔}BS$

Now, H-To-BS communication link messages (refer Figure 2) should be re-arranged from the generic form to the idealized form, as follows:

Generic messages form
  Msg 1: *H_i_* ➔*BS*: <*D, Kidx _Hi_*> (*here, D* = *E_KHi_[HID_i_, ID_BS_, K]*)
  Msg 2: *BS* ➔ *H_i_*: <*Z, K*> (*here, Z* = *E_KHi_[P, N, R2]*)
Idealized messages form
  Msg 1: *H_i_* ➔*BS*: *{HID_i_, ID_BS_, K} _KHi,_, Kidx_Hi_*
  Msg 2: *BS* ➔ *H_i_*: *{P, N, K, R2}_KHi_, K*
*Session Key P* = *h*(*ID_BS_*‖*HID_i_*‖*K*‖*R2*)

To analyze the H-To-BS link, the following assumptions are also needed:

(*C1*) *H-sensor* ***believes*** $BS\overset{\text{KHi}}{↔}H‐\text{sensor}$
(*C2*) *BS* ***believes*** $H‐\text{sensor}\overset{\text{KHi}}{↔}\text{BS}$
(*C3*) *H-sensor* ***believes*** (*BS* ***controls*** *ID_BS_*)
(*C4*) *BS* ***believes*** (*H-sensor* ***controls*** *HID_i_*)
(*C5*) *H-sensor* ***believes*** $BS\overset{P}{↔}H‐\text{sensor}$
(*C6*) *BS* ***believes*** $H‐\text{sensor}\overset{P}{↔}BS$
(*C7*) *BS* ***believes*** $BS\overset{P}{↔}BS$
(*C8*) *H-sensor* ***believes*** (*BS* ***controls*** $BS\overset{P}{↔}H‐\text{sensor}$)
(*C9*) *BS* ***believes*** *H-sensor* ***fresh*** (*K*)
(*C10*) *H-sensor* ***believes*** *BS* ***fresh*** (*R2*)

Now, based on above assumptions and BAN logic rules [39], we will present the H-To-L communication link formal proof, as follows:

H-To-BS correctness proof
  Msg 1: *H-sensor* ➔ *BS*: *{HID_i_, ID_BS_, K}_KHi,_, Kidx_Hi_*
    *D1*) *BS* ***sees*** *{HID_i_, ID_BS_, K}_KHi,_, Kidx_Hi_* //*by Seeing rule*
    *D2*) *BS* ***believes*** (*H-sensor* ***controls*** *HID_i_*)//*C4, D1, Control rule*
    *D3*) *BS* ***believes*** *H-sensor* ***fresh*** (*K*)//*C9, Fresh rule*
    *D4*) *BS* ***believes*** *H-sensor* ***said*** *{P, N, R2}_KHi_*//*Message-meaning rule*
  Msg 2: *BS* ➔ *H-sensor*: *{P, N, K, R2}KH_i_, K*
    *D5*) *H-sensor* ***Sees*** *{P, N, K, R2}_KHi_*//*Seeing rule*
    *D6*) *H-sensor* ***believes*** (*BS* ***controls*** *ID_BS_*)//*C3, D5, Control rule*
    *D7*) *H-sensor* ***believes*** *BS* ***fresh*** (*R2*)//*C10, D5, Fresh rule*
    *D8*) *H-sensor* ***believes*** (*BS* ***controls*** $BS\overset{P}{↔}H_{i}$)//*C8, D5*
    *D9*) *H-sensor* ***believes*** $BS\overset{P}{↔}H‐\text{sensor}$

By performing BAN logic, we have formally verified the H-To-BS communication link goals, *i.e.*, *C5* and *D9* establish a secure and strong session-key (*i.e.*, *P*) between the H-sensor and the base-station. Hence, *C3*, *C4*, *D2*, *D6* and *D9* verify the mutual-authentication between the H-sensor and BS.

## 6. Framework Evaluation

### 6.1. Security Analysis

This sub-section presents the security analysis of the proposed framework. Moreover, it is worth comparing the security services of the proposed scheme with the protocols in [28,30–33,37], since these schemes are specially proposed for heterogeneous sensor networks. Recalling the attack model (section 2.2), an attacker has full control over the wireless channels. It can monitor both communication links (*i.e.*, H-To-L and H-To-BS) and intercept or alter the packets while the packets are in transit. Based on these assumptions, we will analyze the proposed framework, as below:

(1) *Mutual authentication*: In real applications, it is highly desirable that after the sensor deployment, they should prove their legitimacy and maintain the mutual trust during the secure network formation. The proposed scheme achieves mutual authentication for the H-To-L communication link, and for the H-To-BS communication link.
  In the H-To-L link (Figure 2): The L-sensor verifies the authentication of the H-sensor using a *hello* message (*i.e.*, *B′* = *B*). Similarly the H-sensor verifies the authenticity of the L-sensor (*LID_i_*) by decrypting the sub-message <*C*> (*i.e.*, *E_KLi_ [LID_i_*, *Loc_i_*, *R0]*) using key *KL_i_*. Here, the key *KL_i_* is known to the dedicated legitimate nodes (*i.e.*, L-sensor and H-sensor). Furthermore, the L-sensor checks *HID_i_* by decrypting the message <*M*> (*i.e.*, *E_X_ [LID_i_*, *HID_i_*, *R0*, *Q]*) using key *X*. Here the secret key *X* is derived from *KL_i_* and *R0* (*i.e.*, *X*= (*KL_i_⊕ R0*)) and it is only known to the communicating (legitimate) nodes. As we have seen, the mutual authentication takes place between two trusted entities (H-sensor and the L-sensor) and nobody else (*i.e.*, an adversary) can decrypt the messages (*i.e.*, (*E_KLi_ [LID_i_*, *Loc_i_*, *R0]*) and (*i.e.*, *E_X_ [LID_i_*, *HID_i_*, *R0*, *Q]*)) without knowing the secret keys (*i.e.*, *KL_i_* and *X*).
  Similarly, in the H-To-BS link (Figure 3), the BS verifies the authenticity of the H-sensor (*HID_i_*) by decrypting the sub-message <*D*> (*i.e.*, *E_KHi_[HID_i_*, *ID_BS_*, *K]*) using key *KH_i_*. To verify the authenticity of the BS, H-sensor decrypts the message <*Z*> (*i.e.*, *E_KHi_[P*, *N*, *K*, *R2]*) using key *KH_i_* and obtains the following parameters P*, N*, K*, *R2*. It verifies N* = *N*, which proves that the BS is a real entity. Moreover, here, the key *KH_i_* is only known to the communicating H-sensor and the BS.
  It is easy to realize that the intruder cannot impersonate a legal entity in the proposed framework because it performs strong mutual authentication. Hence, the proposed framework maintains the mutual trust for the H-To-L link and the H-To-BS link.
(2) *Strong dynamic session key establishment*: It is clear to see from the H-To-L link (Figure 2), that the proposed framework establishes a strong dynamic (symmetric) session key (*i.e.*, *Q*) after the mutual authentication takes place. The dynamic session key will be used to secure the subsequent H-To-L communications between the H-sensor and the L-sensor. The *Q* will be computed over (=*h*(*R0*‖*R1*‖*LID_i_*‖*HID_i_*)). Here, *R0* and *R1* are the dynamic secrets of L-sensor and H-sensor, respectively. Moreover, the dynamic session key (*Q*) is encrypted in message <*M*> (*i.e.*, *E_X_ [LID_i_*, *HID_i_*, *R0*, *Q]*) using secret key *X* = (*KL_i_ ⨁ R0*)), which is only known to the communicating legitimate nodes. Therefore, an adversary cannot illegally get dynamic session-key.
  Similarly, in the H-To-BS communication link (Figure 3), a strong dynamic session key (*i.e.*, *P*) is established after performing the mutual authentication. The session key (*P*) is a hashed value which is computed over *ID_BS_*, *HID_i_*, *K*, and *R2*. In addition, the *P* is only known to the H-sensor and the BS. Since the dynamic session key (*P*) is encrypted in message (*i.e.*, *E_KHi_[P*, *N*, *K*, *R2]*), an intruder cannot illegally intercept the session key. Hence, strong dynamic session keys (*Q* and *P*) are established in the proposed framework for both the communication links (H-To-L and H-To-BS).
(3) *Message confidentiality*: In general, protocol messages float in the open air, which attracts the attackers. We assume that if an attacker is active in the network from the very beginning, then it can easily capture useful information while the messages are in transit. However, in the proposed protocol, all the useful information (messages) are encrypted (*i.e.*, confidential) in the both communication links (*i.e.*, H-To-L and H-To-BS). The confidential messages are *B* = *h*(*HID_i_*), *C* = *E_KLi_[LID_i_*, *Loc_i_*, *R0]*, *M*= *E_X_[LID_i_*, *HID_i_*, *R0*, *Q]*, *D*=*E_KHi_[HID_i_*, *ID_BS_*, *K]* and *Z* = *E_KHi_[P*, *N*, *K*, *R2]*. All the keys (*i.e.*, *KL_i_*, *X*, *KH_i_*) are only known to the legitimate nodes. Thus, an attacker cannot extract any valuable information from the open air messages.
(4) *Message Freshness*: If the message freshness is not considered properly in the security protocols then confidentiality and authentication have no meaning, so it is desirable that the protocol messages be fresh, *i.e.*, messages are recent, ensuring that no message is replayed and altered. In the proposed scheme, *R0*, *R1* and *K*, and *R2* are dynamic secrets for the L-sensor, H-sensor and BS, respectively, and this ensures that the messages are fresh or recent for every session. Hence the proposed scheme achieves secure message freshness.
(5) *Identity privacy* (*i.e.*, *anonymity*): We assume that if an attacker determines the identity of communicating parties from the wireless packets then he/she can pose many threats (e.g., Sybil attack). Therefore, identity privacy is an important concern in many real-time applications [44,45]. However, the proposed framework takes care of the identity privacy for all the network entities (*i.e.*, L-sensor, H-sensor, and base station). As we can clearly observe from Figures 2 and 3, all nodes' identities are secured, *i.e.*, identities (IDs) and not transmitted as plaintext messages. Consequently, the proposed framework is secure against node privacy threats.
(6) *Man-in-the-middle* (*MITM*) *attack*: In practice, a MITM attack is a kind of active eavesdropping where an attacker can set up an independent connection with the targeted nodes. A MITM makes the network believe that the target nodes are connected with a legitimate node, and by doing so it can control the communication links. As a result, MIMT attacks can pose many threats to real-time applications. However, the proposed scheme is a safeguard against the MITM attack as follows. Assume that a MITM is active in the H-To-L communication link and captures the wireless messages, such as <*B*>, <*Kidx_Li_*, *C*> and <*M*, *R1*>. Indeed, an adversary can easily capture the wireless packets but it cannot read, modify and alter the packets since all the messages are confidential. Similarly, the H-To-BS communication links are kept secure and thus MITM attacks have no option to be successful in the proposed mutual authentication framework.
(7) *Resist replay attack*: In this attack, the adversary first eavesdrops the communication between two communicating entities and then tries to impersonate the legal entity (e.g., sensor/cluster-head/base-station) by simply replaying old messages to the dedicated entity. It is obvious that an adversary can capture some wireless messages and then replay them in later time. For instance:
  (i) In the H-To-L communication link (*c.f.* Figure 2): the adversary captures L-sensor messages (*i.e.*, <*Kidx_Li_*, *C*>) and tries to replay a captured message to the H-sensor again after some time. Since the sub-message *C* has a fresh random number (*i.e.*, R0*), the adversary cannot succeed in replaying the old messages. For every session, R0* is fresh, and is verified with the previously stored values. If R0* is matched with a previously stored session value then the H-sensor will abort the system. Similarly, suppose an adversary captures an H-sensor message (*i.e.*, <*M*, *R1*>) and tries to replay it (the captured message) again after some time to the L-sensor. The attacker will not get succeeded in replaying the old messages because in each session the L-sensor verifies the fresh random number (*i.e.*, R0* = *R0*, if yes then it aborts the system).
  (ii) In the H-To-BS communication link (*c.f.* Figure 3): Likewise (as in the above), the adversary cannot succeed in replaying the old H-sensor messages (*i.e.*, <*D*, *Kidx_Hi_*>) to the BS. The sub-message *D* contains the H-sensor random number (*i.e.*, *K*) and if *K* is matched with the previously stored session then the BS will terminate the system. Similarly, suppose the adversary replays old BS messages <*Z*, *K*> to the H-sensor. The attacker will not succeed because the H-sensor verifies its own generated random number (*i.e.*, K* = *K*) for every session. Consequently, replay attacks are not feasible in the proposed framework.
(8) *Information-leakage attack*: An information-leakage attack is also a kind of active eavesdropping attack where an attacker can leak the protocol valuable information (*i.e.*, *IDs*, *locations*, *etc.*) and thus could be dangerous for many real-time applications (nuclear power plants, *etc*). Nevertheless, this kind of attack cannot harm our scheme. Suppose an eavesdropper captured H-To-L link messages (such as <*Kidx_Li_*, *C*>, <*M*, *R1*>) and H-To-BS messages (such as <*D*, *Kidx_Hi_*> and <*Z*, *K*>) as shown in Figure 2 and Figure 3, respectively. Through these messages the attacker cannot extract any valuable information (*identities*, *locations*, *etc.*) because all the secret information is encrypted using secret keys (*i.e.*, *KL_i_*, *X*, *KH_i_*). In other words, the framework messages are not transmitting as plaintext. Hence an attacker cannot mount an information-leakage attack on the WSN.
  A security functionalities comparison with existing schemes is shown in Table 2. It is clear from this Table that the schemes presented in [28,30–33,37] are designed with limited security services, and none of protocols even discuss the topic of mutual authentication, which is an indispensable security requirement in real WSNs. The schemes in [30,31,37] establish weak session keys which may vulnerable to traffic analysis attacks. In contrast, the proposed framework not only provides mutual authentication between the two devices but it also takes care of other indispensable security services (e.g., dynamic session key, message confidentiality, node privacy, and message freshness) and safeguards against security attacks.

### 6.2. Performance Analysis

In the proposed framework, the computation and communication costs for the H-To-L link are reasonable. Especially, the computation cost incurred at the (resource-constraint) L-sensor is well-suited, since it needs to execute hash functions twice (*B'* = *h*(*HID_i_*) and *Q*), time encryption once (*C* = *E_KLi_[LID_i_*, *Loc_i_*, *R0]*), and time decryption once (*D_X_′[M]*), as shown in the H-to-L link (Figure 2). On the other hand, the H-sensor has more resources than the L-sensor and can perform more complex computations. Therefore we elaborate on analyzing all the memory required and the processing time (which includes hash function, encryption and decryption operation) for L-sensors. Consider a MicaZ node that has a single processor board (MPR2400 based on ATmega128L), 4 Kb of RAM, 128 Kb of ROM and uses a CC2420 radio [9]. In [51,57], it has been demonstrated that Skipjack encryption/ decryption is a most energy-efficient cryptosystem. The Skipjack implementation requires 0.6 Kb of RAM and 10 Kb of ROM (*i.e.*, memory space) on a MicaZ mote. The estimated processing time for (Skipjack) encryption and decryption operations are 0.22 ms (millisecond) and 0.22 ms, respectively (refer Table 1 and 5 in [57]). Moreover, it consumes 5.52 μj of energy in encryption and 5.52 μj of energy in decryption. In addition, Lee *et al.* demonstrated that a hash function (HMAC-MD5) gives 128 bits of digest and it requires 0.1 KB of RAM and 32Kb of ROM on a Micaz platform (refer Table 7 in [57]). Table 3 shows the computation cost of the proposed framework, *i.e.*, for H-To-L links, which is significantly affordable to the L-sensors.

The computation cost for the H-To-BS links is not a prime concern since the H-sensor and BS are resource rich and it is assumed that both of them (H-sensor and BS) can compute more complex computations than the L-sensor. Nevertheless, Table 3 also summarizes the computation cost for the H-To-BS link, where the H-sensor computes hash functions two times, encryption one time and decryption one time. Computation costs incurred at the BS are similar.

In the authentication protocols, the communication cost is an extra overhead that depends on how many bits/bytes are being transferred and how many messages are being exchanged during the protocol execution. For the sake of simple communication overhead, which is incurred at the sensor side, we consider 2 bytes for each id-length (*i.e.*, *HID_i_*, *LID_i_*, *and BS_ID_*), 16 bytes for each hash digest, 2 bytes for each nonce/random-number, and 1 byte each for key index identifier.

- In the H-To-L link, two receptions and one transmission are required at the L-sensor, as shown in Figure 2. The total length of two received messages (*i.e.*, *hello* message, and *M* = *E_X_ [LID_i_*, *HID_i_*, *R0*, *Q]*, *R1*) and one transmitted message (*i.e.*, <*Kidx_Li_*, *C*>) is about 46 bytes, roughly.
- Likewise, as shown in Figure 3, the H-To-BS link requires one transmission (*i.e.*,<*D*, *Kidx_Hi_*>) and one reception (*i.e.*, <*Z*, *K*>) at the H-sensor side. The total length of messages being transmitted is roughly 45 bytes.

Moreover, with reference to [52], it has been considered that the message transmission energy consumption rates are roughly over three orders of magnitude greater than the energy consumption rates for message computing. Table 4 summaries the communication cost of our proposed framework, *i.e.*, H-To-L links require three message exchanges, as shown in Figure 2; and H-To-BS links require two message exchanges (as shown in Figure 3).

## 7. Discussion

In mission-critical WSN-based applications, sensors are always deployed strategically, in e.g., organic compound monitoring, nuclear plant monitoring, body area networks, gas plant monitoring, structural health monitoring and many more (such as described in [1,2,4,5,7,41,42,55]). To maintain the mutual trust between the network devices an efficient and adaptive mutual authentication framework which can protect and make safe the application/network from unauthorized access is highly required from the beginning of network deployment [58–60]. By performing the mutual authentication, two legitimate parties (e.g., sensors, cluster heads and/or base stations) can establish a trust using their legitimate identities. Otherwise, it is difficult to protect the application data from unauthorized access and message modifications, and from security attacks/threats, e.g., replay attacks, eavesdropping attacks, message leakage attacks and man-in-the-middle attacks. Consider the following application scenarios:

(i) In body area network (BAN) applications, a group of BAN devices must be associated with an intended patient, lest the wrong medical data be collected. In BANs, it is highly required that each medical sensor must be mutually authenticated to the BAN coordinator device to form a trusted and safe BAN.
(ii) Likewise, in a smart grid where wireless sensors are deployed into the grid, if mutual authentication is not taken into account then the attackers can pose man-in-the-middle threats/attacks to the smart grid. Moreover, to disturb the smooth functioning of the network, the attacker can impersonate legal nodes which may cause denial-of-service attacks. Therefore, an adequate mutual authentication mechanism between all entities of the network should be provided to confront these threats.

The fundamental differences between the proposed framework and the existing literature are the following:

- The proposed framework performs the mutual authentication and establishes a dynamic session key between the two communicating entities (*i.e.*, L-To-H link and H-To-BS link), while the existing schemes fail to perform the mutual authentication between the communicating entities.
- Another concern of real-time applications are privacy issues (*i.e.*, node privacy and content privacy) [44]. However, the advantage of the proposed framework over existing schemes is the node privacy. In the proposed scheme the node identity is protected within the encrypted packets, while the proposals in [28,30–33] leak the node identities to the attacker (*i.e.*, node privacy is breached). Hence it is easy to say that the proposed framework fulfills the node privacy and the content privacy requirements, which are not exposing the network to a global outside adversary.
- In practice, the secure links can be compromised if a sensor is physically captured by an adversary. The adversary can extract all the stored keys from the compromised node and may try to connect to the remaining non-compromised nodes in the network. It is very difficult to avoid the physical capturing of nodes unless they are either tamper-resistant or guarded. Nevertheless, in proposed framework, the H-sensors are tamper-proof but the L-sensors are deployed in an application environment (open/indoors) and are neither tamper-proof nor guarded. Thus, L-sensors are directly available to the node capture attacks that can disturb the communication links. In the proposed framework, each L-sensor is preloaded with a single key which is only shared with its H-sensor. Therefore a single compromised node will not disturb the working of other non-compromised nodes. In contrast, in other options an L-sensor is preloaded with numbers of keys (e.g., in [28,30,37]), which may pose more harm to non-compromised nodes, if the L-sensor is compromised by an adversary.

## 8. Conclusions

In this paper, we have introduced an efficient and adaptive mutual authentication framework which leverages the concept of symmetric cryptography. As we have seen, the proposed scheme couples many indispensable security services (such as, mutual authentication and session key establishment, message confidentiality, node privacy, and message freshness) and safeguards against security attacks. In addition, we have formally verified the correctness of the proposed protocols using BAN logic. An extensive security analysis reveals that the proposed framework not only provides many security services, but it is a safeguard against possible security attacks (such as man-in-the-middle attacks, replay attacks, impersonation attacks and information-leakage attacks) as compared to other existing schemes. Consequently, the proposed framework achieves efficiency (in terms of computation and communication costs), and is practical for real-world tiny wireless sensor networks.

## Figures and Tables

**Table 1.** Notations and descriptions.

**Notations**	**Descriptions**
*BS*, *ID_BS_*	A base-station and its identity
*H-Sensor*, *HID_i_*	High-capacity sensor (Cluster-head) and identity of *i^th^* H-sensor
*L-Sensor*, *LID_i_*	Low-capacity sensor and identity of *i^th^* L-sensor
*KPH*, *KPL*	Key pool for H-sensors, and for L-sensors
*KH_i_*	Pool key for *i^th^* H-sensor, *i* ∈ *{1*,*2*,*3*…*H}*
*Kidx_Hi_*	Key index of *KH_i_*, *i* ∈ *{1*,*2*,*3*,…*H}*
*KL_i_*	Pool key for *i^th^* L-sensor, *i* ∈ *{1*,*2*,*3*…*L}*
*Kidx_Li_*	Key index of *KL_i_*, *i* ∈ *{1*,*2*,*3*,…*L}*
*Loc_i_*	Location of *i^th^* sensor
*E_x_[]*	Symmetric encryption and decryption using key *x*
*D_x_[]*	Symmetric decryption using key *x*
*h*(.)	One way hash function, e.g., *SHA-1*
*⨁*, ‖	Bit-wise *XOR* operation, and concatenation function

**Table 2.** Security services comparisons with the existing protocols.

**Security Services**	**[28]**	**[30]**	**[31]**	**[32]**	**[33]**	**[37]**	**Proposed**
S1	N	N	N	N	N	N	**Y**
S2	N	W	W	N	N	W	**Y**
S3	N	N	Y	Y	Y	Y	**Y**
S4	N	W	N	Y	N	N	**Y**
S5	N	N	N	N	N	P	**Y**
S6	Y	N	N	W	N	Y	**Y**
S7	Y	N	Y	Y	N	Y	**Y**
S8	N	N	N	N	N	P	**Y**
S9	N	N	N	N	N	P	**Y**
S10	N	Y	N	Y	Y	Y	**Y**

S1 = Mutual authentication; S2 = Strong dynamic session key; S3 = Message confidentiality; S4 = Message freshness; S5 = Identity privacy; S6 = Key revocation; S7 = New node addition; S8= Secure against MITM; S9 = Secure against information-leakage attack; S10: Secure against replay attack; N = No; Y = Yes; W = Weak; and P = Partial.

**Table 3.** Computation cost for H-To-L and H-To-BS link operations.

**Cryptographic Operations**	**H-To-L**		**H-To-BS**	

	**H**	**L**	**H**	**BS**
Hash	2	2	2	2
Encryption	1	1	1	1
Decryption	1	1	1	1

**Table 4.** Communication cost for H-To-L and H-To-BS links.

	**H-To-L**	**H-To-BS**
No. of messages exchanged	3	2
